# Ulinastatin treatment mitigates glycocalyx degradation and associated with lower postoperative delirium risk in patients undergoing cardiac surgery: a multicentre observational study

**DOI:** 10.1186/s13054-025-05296-9

**Published:** 2025-01-29

**Authors:** Xiao Ran, Tingting Xu, Jieqiong Liu, Shaobing Yang, Fang Luo, Rongxue Wu, Juan Tan, Hang Ruan, Qin Zhang

**Affiliations:** 1 Department of Critical Care Medicine, Tongji Hospital, Tongji Medical College, Huazhong University of Science and Technology, Wuhan, 430000 China; 2 Department of Emergency Medicine, Tongji Hospital, Tongji Medical College, Huazhong University of Science and Technology, Wuhan, 430000 China; 3 Department of Anesthesiology, Hubei Key Laboratory of Geriatric Anesthesia and Perioperative Brain Health, Wuhan Clinical Research Center for Geriatric Anesthesia, Tongji Hospital, Tongji Medical College, Huazhong University of Science and Technology, 1095# Jiefang Ave, Wuhan, 430030 China; 4 Department of Biological Sciences Division - Cardiology, University of Chicago, Chicago, IL USA

**Keywords:** Cardiac surgery, Postoperative delirium, Ulinastatin, Glycocalyx degradation, Mediation effect, Critical care

## Abstract

**Background:**

Ulinastatin (UTI), recognized for its anti-inflammatory properties, holds promise for patients undergoing cardiac surgery. This study aimed to investigate the relationship between intraoperative UTI administration and the incidence of delirium following cardiac surgery.

**Methods:**

A retrospective analysis was performed on a retrospective cohort of 6,522 adult cardiac surgery patients to evaluate the relationship between UTI treatment and the incident of postoperative delirium (POD) in patients ongoing cardiac surgery. This was followed by a prospective observational cohort study of 241 patients and an in vitro study to explore the findings and the potential role of UTI in preventing cardiac ischemia–reperfusion induced glycocalyx degradation.

**Results:**

Both univariate and multivariate logistic regression analyses in retrospective cohort indicated that intraoperative administration of UTI was associated with a significant lower risk of POD among cardiac surgery patients, a finding confirmed through employing propensity score matching. The subsequent prospective observational cohort further supported these findings (adjusted Odds Ratio = 0.392, 95% CI: 0.157–0.977, *P* = 0.044). Furthermore, UTI mitigated glycocalyx degradation, as demonstrated by in vitro study.

**Conclusions:**

UTI administration may mitigate glycocalyx degradation, potentially lowering the risk of POD in cardiac surgery patients, offering valuable insights for future interventions to prevent POD and enhance patient outcomes.

*Trial registration number* ClinicalTrials.gov (No. NCT06268249). Retrospectively registered 4 February 2024.

**Supplementary Information:**

The online version contains supplementary material available at 10.1186/s13054-025-05296-9.

## Introduction

Postoperative delirium (POD), an acute cognitive dysfunction, is a prevalent, serious, and under-recognized complication following cardiac surgery [1–3]. POD is a significant concern given its prevalence rate of 12% to 55% and its correlation with negative outcomes including long-term cognitive decline, extended hospitalization, and heightened healthcare expenses [4–8]. Currently, there is no standardized treatment for POD [9], underscoring the necessity of implementing a protocol for prompt detection and intervention in cardiac surgery patients.

The glycocalyx is a layer that coats the luminal surface of vascular endothelial cells [10]. Following cardiac surgery, pro-inflammatory factors are triggered, leading to glycocalyx degradation. This degradation results in endothelial cell dysfunction, impairing vascular diastolic function and increasing vascular permeability, eventually causing leakage of the blood–brain barrier (BBB) [11, 12]. While the pathogenesis of POD remains uncertain, it has been associated with neuroinflammation, neuronal metabolic disturbances, and BBB dysfunction [13–16]. Hyaluronic acid (HA), a major component of the glycocalyx, increases in plasma levels in response to glycocalyx degradation [17]. Plasma HA levels are recognized as biomarkers of endothelial glycocalyx damage in conditions such as sepsis and ischemia–reperfusion (I/R) injury [18]. Hence, it is hypothesized that cardiac surgery-induced glycocalyx degradation may be linked to POD and could be detected through plasma HA levels.

Ulinastatin (UTI), a wide-ranging protease inhibitor derived from human urine, possesses anti-inflammatory and cytoprotective properties. It is commonly employed in the management of inflammatory conditions and cardiac and vascular surgeries. While UTI treatment in surgical patients has shown promise in reducing the incidence of postoperative cognitive dysfunction, the precise underlying mechanisms remain unclear [19, 20]. Previous research, including studies from our group, suggests that UTI may confer benefits to individuals undergoing cardiac surgery by dampening inflammation and reinforcing endothelial barrier function [21–24].

Building on this foundation, we hypothesize that UTI administration during cardiac surgery could potentially attenuate glycocalyx degradation and lower the incidence of POD. The study is structured in three steps: Step 1 is a multicenter retrospective analysis assessing the link between intraoperative UTI treatment and POD risk in cardiac surgery patients. Step 2 involves a prospective cohort study investigating the relationship between intraoperative UTI administration, glycocalyx degradation 24 h post-cardiac surgery, and POD. Step 3 encompasses an in vitro study investigating the effect of UTI on glycocalyx degradation and the enhancement of endothelial barrier function following cardiac surgery.

## Materials and methods

### Study design

**Fig. 1:**
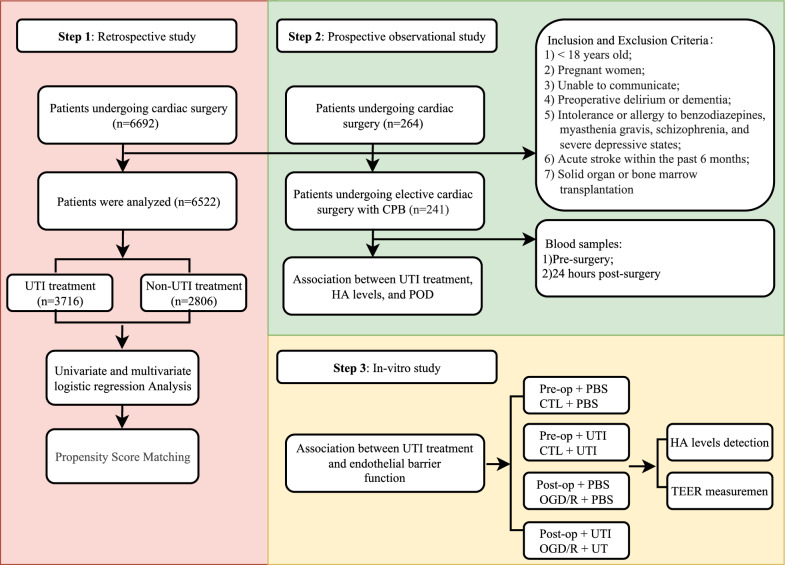
Flowchart of study design. Note: During the retrospective study phase, the study evaluated the effect of administering UTI during surgery on POD occurrence, as well as the identification of potential risk or protective factors. Subsequently, an independent prospective cohort study and an in vitro study were initiated to verify the findings and elucidate the potential role of UTI in mitigating glycocalyx degradation to prevent POD in cardiac surgery patients. Abbreviations: UTI, ulinastatin; POD, postoperative delirium; PSM, propensity score matching; CPB, cardiopulmonary bypass

This study investigated the association between intraoperative administration of UTI and the incidence of POD after cardiac surgery. It comprised three key components: an innovative retrospective study, a prospective cohort study, and an in vitro study. The study design was depicted in Fig. 1.

### Step 1: retrospective study

The study population for the derived cohort included individuals who underwent elective cardiac surgery at the three medical centres of Tongji Hospital from January 2015 to December 2022. The patients were excluded from the study if they (1) were < 18 years old; (2) were pregnant women; (3) were unable to communicate effectively due to visual, auditory, or language impairments; (4) had preoperative delirium or dementia; (5) had conditions including intolerance or allergy to benzodiazepines, myasthenia gravis, schizophrenia, and severe depressive states; (6) had history of acute stroke within the past 6 months; (7) received solid organ or bone marrow transplantation.

The presence of POD within 7 days after surgery was designated as the primary outcome. The three medical centres of Tongji Hospital routinely conduct delirium assessments for patients after surgery. Postoperative patients who have undergone heart surgery undergo regular monitoring in the intensive care unit (ICU). They are typically assessed every 6 to 8 h post-surgery using standardized scoring systems. The assessment findings are meticulously recorded in both nursing and medical records for comprehensive documentation and monitoring of patient progress.

To identify POD, diagnostic procedures from previous studies were used [25]. Two scenarios were considered: (1) a POD diagnosis already documented was deemed as recognized delirium and accepted as the accurate diagnosis, and (2) if no POD diagnosis was initially recorded, data collectors retrospectively assessed the clinical records for signs of delirium. The criteria used for retrospective POD diagnosis were aligned with the adapted Statistical Manual of Mental Disorders, Fifth Edition (Supplementary Delirium Diagnostic Criteria) [25]. Collected data included demographic characteristics, chronic comorbidities, intraoperative administration of UTI, POD status, preoperative vital signs, preoperative shock, infection and anemia. Data collection and disease diagnosis were conducted independently by two clinicians specialized in delirium diagnosis to uphold data integrity and precision. Subsequently, the researchers cross-validated the gathered data to verify consistency and accuracy. In cases of discrepancies or divergences between collectors, a neutral third party was engaged to mediate and reconcile any inconsistencies in data collection.

### Step 2: prospective observational study

The prospective observational study, comprising patients undergoing elective cardiac surgery with cardiopulmonary bypass (CPB) such as open-heart valve repair, or replacement, was prospectively gathered from April 2023 to December 2023. A total of 241 patients were included, with follow-up assessments conducted up to 7 days postoperatively. The inclusion and exclusion criteria in the validation were the same as the derivation cohort.

At baseline, all patients underwent preoperative assessments the day before their surgical procedures, encompassing the collection of sociodemographic data, medical histories, comorbidities, and evaluation of cognitive and physical functions. Data collected in the prospective observational cohort comprised demographic characteristics, chronic comorbidities, intraoperative administration of UTI, postoperative consciousness status, preoperative inflammatory and biochemical markers.

UTI improves vascular permeability and microcirculation by mitigating damage to vascular endothelial cells caused by oxidative and inflammatory injuries [24]. At Tongji Hospital, UTI treatment during surgery has been adopted by some cardiothoracic surgeons. According to our institutional guidelines, its administration is commonly indicated in several clinical scenarios: 1. treatment of acute pancreatitis, 2. management of shock, 3. treatment of sepsis, 4. severe pneumonia, 5. acute respiratory distress syndrome, 6. valve surgery, 7. major vessel surgeries, and 8. coronary artery bypass grafting. The decision to employ UTI during surgical procedures is influenced by factors including the availability of the medication, anesthesiologist prescribing preferences, and their assessment of the surgical situation—particularly if a prolonged surgical duration or heightened inflammatory response is anticipated.

Follow-up assessments were done daily within seven days postoperatively by physicians trained in the Postoperative Delirium Assessment Training System using the Confusion Assessment Method for the Intensive Care Unit (CAM-ICU) to determine the presence of delirium [26–28]. Inflammatory and biochemical markers were collected within 24 h postoperatively. The primary outcome in this cohort was the incidence of POD. Secondary outcomes included hospital length of stay, duration of surgery, postoperative HA levels, HA concentration difference, and lactate.

### HA levels detection

In step 2 of the study, plasma samples were collected before and 24 h after cardiac surgery for the assessment of HA levels. Furthermore, in Step 3, HA levels were evaluated in the cell culture medium in vitro. Quantification of HA levels was performed using an ELISA kit (HM10597, Bio-swamp, Wuhan, China) according to the manufacturer’s instructions. The concentration of HA in the samples was calculated based on the absorbance values obtained from the standard curve. The remaining plasma samples from both preoperative and postoperative time points were subsequently stored at −80 °C.

### Step 3: in vitro study

The in vitro experiment was divided into two parts. In the first part, to simulate the endothelial ischemia–reperfusion injury associated with cardiac surgery and its potential role in inducing POD, HUVECs were exposed to the plasma of POD patients, both in the preoperative group (Pre-op Group) and the postoperative group (Post-op Group). Concurrently, UTI or phosphate-buffered saline (PBS) was administered to evaluate whether UTI could reduce HA levels and improve endothelial barrier function. The cells were categorized into four groups: (1) Pre-op Group: HUVECs exposed to 100 µl/ml of preoperative plasma from POD patients with vehicle control (PBS); (2) Pre-op + UTI Group: HUVECs exposed to 100 µl/ml of preoperative plasma from POD patients with UTI (3000 U/ml); (3) Post-op Group: HUVECs exposed to 100 µl/ml of postoperative plasma from POD patients with vehicle control (PBS); (4) Post-op + UTI Group: HUVECs exposed to 100 µl/ml of postoperative plasma from POD patients with UTI (3000 U/ml). Plasma samples from both preoperative and postoperative phases were collected from the same POD patients in Step 2. The HA levels and transendothelial electrical resistance (TEER) of the cells in the four groups were measured.

In the second part, the HUVECs were subjected to oxygen–glucose deprivation/recovery (OGD/R) to establish an in vitro model of ischemia–reperfusion injury, which was utilized to assess whether UTI alleviated the increase in HA levels and the disruption of endothelial barrier integrity caused by ischemia–reperfusion injury. Thus, HUVECs were divided into the following groups: (1) CTL Group: HUVECs did not receive OGD/R and were administered vehicle control (PBS); (2) CTL + UTI Group: HUVECs did not receive OGD/R and were administered UTI (3000 U/ml); (3) OGD/R Group: HUVECs received vehicle control (PBS) prior to OGD/R. (4) OGD/R + UTI Group: HUVECs were administered UTI (3000 U/ml) prior to OGD/R. The HA levels and TEER were the primary outcomes in step 3.

### Cell culture

HUVECs were purchased from the American Type Culture Collection (ATCC) in Manassas, VA, USA. They were cultured in Medium 199, which contained 20% fetal bovine serum (FBS), 0.1% glutamine, 0.01% heparin, 0.01% of 50 mcg/ml endothelial cell growth supplement, and 100 U/ml streptomycin and penicillin. The cells were maintained at 37 °C in a humidified atmosphere with 5% CO_2_. The culture medium was refreshed every 48 h for a period of 10 to 14 days, until the cells reached 60–70% confluence.

### Oxygen–glucose deprivation/reoxygenation (OGD/R)

During cardiac surgery, I/R injury induces damage to endothelial cells, which in turn triggers the release of proinflammatory factors and enhances vascular permeability, ultimately resulting in blood–brain barrier BBB dysfunction. This dysfunction permits the infiltration of various inflammatory mediators into the brain, leading to the development of POD [29, 30]. To simulate this condition in an experimental framework, HUVECs were employed as an in vitro model [31–33]. Initially, the cells were cultured in a glucose- and serum-free medium and exposed to hypoxic conditions at 37 °C, using a gas mixture of 5% CO_2_ and 95% N2 for 8 h to induce OGD. Following the OGD phase, the culture medium was replaced with standard medium to restore normal glucose levels (5 mM) and oxygen levels (21%). The cells were then subjected to reperfusion for a period of 24 h.

### TEER measurement

TEER was measured to assess changes in endothelial permeability using an automated impedance sensing system (ECIS; Applied Biophysics, Troy, NY) [34]. HUVECs were seeded at a density of 60,000 cells/cm^2^ in each well of the manufacturer’s eight-well electrode slide (8W10E). The ECIS measurement utilized the multiple frequency/time (MFT) settings to capture impedance values across a wide range of frequencies.

### Statistical analysis

For continuous variables, when the data met the normal distribution and homogeneity of variance test, t-test was employed for two-group comparison. When the data met the normal distribution but not the homogeneity of variance test, the Welch t-test was used for two-group comparison. When the data did not meet the normal distribution, Wilcoxon test was used for two-group comparison. The chi-square test was used to compare the categorical variables between groups. Continuous variables were reported as medians and interquartile range (IQR). Categorical variables were reported as frequencies and percentages. Statistical analyses were performed using Stata 17.0 software (Stata Corp LP, College Station, TX). A bilateral *P*-value of less than 0.05 was considered statistically significant.

### Controls for confounders and subgroup analyses

In step 1 of this study, comprehensive statistical analyses were conducted, encompassing univariate logistic analysis to examine individual variables, multivariate logistic analysis to explore the combined effects of multiple variables, and propensity score matching (PSM) to minimize the impact of potential confounders and enhance the reliability of the study findings. Univariate logistic regression analysis was conducted to assess the association of each potential risk factor with the outcome variable. Factors with a statistically significant association (*P* < 0.1) were further evaluated in the multivariate logistic regression analysis in the retrospective cohort. Initially, the covariates most predictive of UTI treatment were selected using the “psestimate” command. The propensity score for each patient was then estimated using a logistic regression model, followed by 1:1 nearest neighbor matching based on the selected covariates. The quality of the matching was evaluated using the standardized mean difference (SMD), with an SMD value of less than 0.1 indicating good balance. PSM was performed to further explore the association between intraoperative administration of UTI and POD in the retrospective cohort. The impact of UTI in various subgroups was assessed based on patients’ age, gender, height, weight, and comorbidities, with interactions analyzed accordingly. The correlation analysis and sample size calculations for Step 2 of this study are detailed in the Supplementary Correlation Analysis and Sample Size.

### Mediation effects analysis

Mediation analysis was conducted to examine the potential mediating role of HA in the relationship between UTI and POD. We followed the steps outlined by “KHB” methods to assess mediation effects [35]. Firstly, we examined the association between HA, UTI and POD using crude model Subsequently, we investigated the relationship between UTI and POD while controlling for independent variable. Finally, we utilized the “medeff” method to estimate the indirect effect and to determine the significance of the mediating pathway. Statistical significance was set at *P* < 0.05.

### Analysis of in vitro study

In step3, data shown as data were presented as median ± interquartile, and statistical significance was determined using one-way ANOVA with Tukey’s post-hoc test or two-tailed log-rank test.

## Results

### Demographic and clinical characteristics of the retrospective cohort

Among the 6692 screened cardiac surgery patients, 6522 individuals met the eligibility criteria and were included in the analysis cohort. Detailed demographic and clinical characteristics of the study population can be found in Table 1. The median age of the patients was 60 years (IQR: 51–68), with males accounting for 62.9% of the cohort. Specifically, 3,716 patients received intraoperative UTI treatment, while 2,806 did not. Interestingly, those who underwent UTI treatment during surgery exhibited a higher prevalence of hypertension and diabetes mellitus. Moreover, the majority of patients (83.8%) underwent CPB, with a significantly higher proportion of patients receiving intraoperative UTI treatment compared to those who did not (87.5% vs. 79.0%, *P* < 0.001). Remarkably, the incidence of POD in patients who received intraoperative UTI treatment was notably lower than in those who did not (11.3% vs. 15.6%, *P* < 0.001).

**Table 1:** Demographic and clinical characteristics of the retrospective cohort

Characteristics	Total (n = 6522)	UTI treatment (n = 3716)	Non-UTI treatment (n = 2806)	*P*-value
Age, years	60 (51–68)	59 (51–67)	61 (52–69)	< 0.001
Weight, kg	63 (55–72)	64 (55–72)	63 (55–72)	0.205
Height, cm	167 (160–171)	167 (160–171)	166 (160–170)	0.244
*Gender, n (%)*				
Male	4104 (62.9)	2357 (63.4)	1747 (62.3)	0.333
Female	2418 (37.1)	1359 (36.6)	1059 (37.7)	
*Chronic comorbidities, n (%)*				
Hypertension	2325 (35.6)	1398 (37.6)	927 (33.0)	< 0.001
Diabetes mellitus	526 (8.1)	323 (8.7)	203 (7.2)	0.032
Respiratory diseases	39 (0.6)	24 (0.6)	15 (0.5)	0.564
Renal dysfunction	79 (1.2)	44 (1.2)	35 (1.2)	0.817
Tumour	74 (1.1)	49 (1.3)	25 (0.9)	0.106
History of stroke	193 (3.0)	110 (3.0)	83 (3.0)	0.996
History of surgical	1417 (21.7)	782 (21.0)	635 (22.6)	0.124
*Preoperative*				
Shock, n (%)	71 (1.1)	44 (1.2)	27 (1.0)	0.393
Infection, n (%)	1686 (25.9)	956 (25.7)	730 (26.0)	0.792
Anemia, n (%)	2080 (31.9)	1220 (32.8)	860 (30.6)	0.061
Cardiac surgery with CPB, n (%)	5468 (83.8)	3252 (87.5)	2216 (79.0)	< 0.001
*Primary outcome*				
POD, n (%)	857 (13.1)	419 (11.3)	438 (15.6)	< 0.001

Continuous variables were presented as median and interquartile range. Categorical variables were reported as number (proportions).

*UTI* Ulinastatin, *CPB* Cardiopulmonary bypass, *POD* Postoperative delirium

### Association between UTI treatment and reduced risk of POD among cardiac surgery patients in the retrospective cohort

In the retrospective cohort analysis presented in Table 2, involving 6522 cardiac surgery patients, various factors were assessed for their correlation with POD through both univariate and multivariate logistic regression analyses. The univariate logistic regression analysis identified older age, higher weight, male, chronic comorbidities such as hypertension and respiratory diseases, undergoing CPB, taller stature, shock, infection and anemia as risk factors for POD. Collinearity assessment indicated no issues among these variables during the multivariate analysis (Supplementary Table S1). The independent protective effect of intraoperative UTI treatment against POD was confirmed in the retrospective cohort, with a statistically significant odds ratio (OR) of 0.619 (95% confidence interval (CI): 0.531–0.722, *P* < 0.001) in the multivariate logistic regression analysis. To explore the findings, PSM was performed on the retrospective cohort (Supplementary Fig. S1). In the PSM cohort, patients treated for UTI during cardiac surgery had a lower incidence of POD (10.7% vs 15.9%, *P* < 0.001) (Supplementary Table S2). For evaluating potential clinical diversity, interaction and stratification analyses were performed. Across all diverse subgroups, UTI was observed to be protective against the development of POD (All OR < 1, Supplementary Fig. S2). Collectively, these findings suggest that UTI could potentially be an intraoperative pharmacological intervention to prevent POD in cardiac surgery patients.

**Table 2:** Univariate and multivariate logistic regression analysis of postoperative delirium in the retrospective cohort

Characteristics	Univariate analysis		Multivariate analysis	
	OR (95%CI)	*P*-value	OR (95%CI)	*P*-value
Age, years	1.016 (1.010–1.023)	< 0.001	1.029 (1.022–1.037)	< 0.001
Weight, kg	1.031 (1.025–1.037)	< 0.001	1.031 (1.023–1.039)	< 0.001
Height, cm	1.022 (1.013–1.031)	< 0.001	0.980 (0.965–0.995)	0.010
*Gender, n (%)*				
Male	1.595 (1.362–1.869)	< 0.001	1.245 (0.987–1.570)	0.064
*Chronic comorbidities, n (%)*				
Hypertension	1.601 (1.385–1.851)	< 0.001	1.183 (1.008–1.389)	0.040
Diabetes mellitus	0.980 (0.751–1.278)	0.880		
Respiratory diseases	2.618 (1.298–5.277)	0.007	2.970 (1.405–6.276)	0.004
Renal dysfunction	1.070 (0.564–2.032)	0.836		
Tumour	0.799 (0.382–1.671)	0.552		
History of stroke	1.078 (0.713–1.629)	0.723		
History of surgical	1.030 (0.867–1.225)	0.735		
*Preoperative*				
Shock, n (%)	4.689 (2.905–7.569)	< 0.001	3.242 (1.928–5.455)	< 0.001
Infection, n (%)	3.596 (3.102–4.169)	< 0.001	3.020 (2.575–3.541)	< 0.001
Anemia, n (%)	1.345 (1.159–1.562)	< 0.001	1.339 (1.141–1.573)	< 0.001
Cardiac surgery with CPB, n (%)	2.363 (1.833–3.047)	< 0.001	3.107 (2.374–4.067)	< 0.001
UTI treatment, n (%)	0.687 (0.595–0.793)	< 0.001	0.619 (0.531–0.722)	< 0.001

Adjusted by age, weight, height, gender, hypertension, respiratory diseases, preoperative shock, preoperative infection, preoperative anemia, cardiac surgery with CPB and UTI treatment.

*CPB* Cardiopulmonary bypass, *UTI* Ulinastatin

### UTI treatment linked to lower HA levels and reduced POD incidence in the prospective observational Cohort

**Fig. 2:**
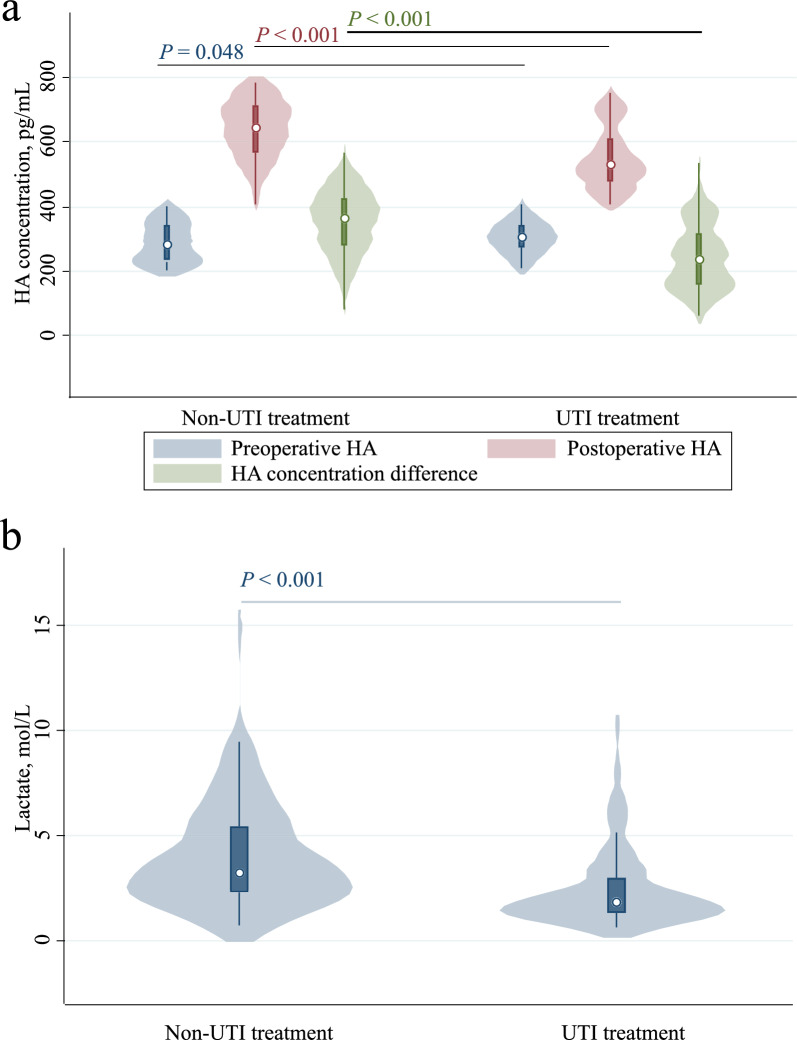
Analysis of the association between independent variables and postoperative delirium (POD). **a** Comparison of preoperative hyaluronic acid (HA), postoperative HA, and HA concentration difference between the two groups of patients. **b** Comparison of lactate levels between the two groups

The demographic characteristics of the prospective observational cohort, detailed in Supplementary Table S3, included 241 patients, with 99 receiving intraoperative UTI treatment and 142 not. Notably, patients who underwent UTI treatment during surgery showed a significantly reduced risk of POD (7.1% vs. 16.2%, OR = 0.394, 95% CI: 0.162–0.957, *P* = 0.040; adjusted OR = 0.392, 95% CI: 0.157–0.977, *P* = 0.044, Table 3). Postoperative levels of HA were notably lower in patients treated for UTI during cardiac surgery compared to untreated patients (median, 530 pg/ml, IQR: 478–610, vs. median, 644 pg/ml, IQR: 567–711, *P* < 0.001), even with lower preoperative HA levels in the latter group (median, 305 pg/ml, IQR: 273–342 vs. median, 282 pg/ml, IQR: 231–341, *P* = 0.048). Further analysis revealed a significantly lower increase in postoperative HA levels for patients treated for UTI during cardiac surgery compared to those without UTI treatment, with the HA concentration difference also being markedly lower in the UTI-treated patients (median, 236 pg/ml, IQR: 161–320, vs. median, 364 pg/ml, IQR: 281–428, *P* < 0.001) (Fig. 2a and Supplementary Table S3). These findings suggest that intraoperative UTI treatment may lower HA levels and decrease the risk of POD. In addition, patients treated for UTI during cardiac surgery had lower postoperative lactate levels compared to untreated patients (median, 1.8 mmol/L, IQR: 1.3–3.0, vs median, 3.2 mmol/L, IQR: 2.3–5.4, P < 0.001) (Fig. 2b and Supplementary Table S3, S4). Supplementary Figs. S3 and S4 illustrate the association of levels of HA and lactate with POD, suggesting that postoperative HA levels could potentially be utilized as a biomarker for predicting POD.

**Table 3:** Impact of ulinastatin treatment on clinical outcomes

Outcomes	OR/Coef (95%CI)	*P*-value	adjusted OR/Coef (95%CI)	*P*-value
*Primary outcomes*				
POD	0.394 (0.162–0.957)	0.040	0.392 (0.157–0.977)	0.044
*Secondary outcomes*				
Lac, mmol/L	−1.493 (−2.049–0.937)	< 0.001	−1.557 (−2.121–0.992)	< 0.001
Postoperative HA, ng/mL	−0.092 (−0.116–0.068)	< 0.001	−0.097 (−0.122–0.073)	< 0.001
HA concentration difference, ng/mL	−0.106 (−0.132–-0.080)	< 0.001	−0.097 (−0.122–0.073)	< 0.001
Duration of surgery, hour	−0.340 (−0.778–0.098)	0.127	−0.291 (−0.732–0.149)	0.194
Hospital length of stay, days	0.418 (−2.286–3.121)	0.761	0.398 (−2.387–3.183)	0.779

Adjusted by preoperative HA, preoperative troponin I, preoperative lactate dehydrogenase

*POD* Postoperative delirium, *Lac* Lactate, *HA* Hyaluronic acid

### HA concentration as a mediator in the relationship between UTI treatment and reduced risk of POD in cardiac surgery patients

To explore the relationship between intraoperative administration of UTI, HA concentration, and POD, a mediation effect analysis was conducted using the Karlson–Holm–Breen method. The result revealed that intraoperative UTI treatment reduced the probability of POD by 10.3%. After adjusting for HA concentration difference, the probability of POD decreased to 0.9%, indicating that the influence of intraoperative UTI treatment on reducing POD was partially mediated by its capacity to lower HA concentration difference levels (Supplementary Table S5 and Fig. S5). The presence of mediating effects was confirmed in both the KHB mediated effects analysis model, adjusted for various confounders, and the “medeff” mediated effects model (Supplementary Table S6-9). The Directed Acyclic Graph clearly illustrated the indirect effect of the intraoperative administration of UTI on the POD through the HA concentration difference, while also depicting the direct effect of the intraoperative administration of UTI on the POD (Supplementary Fig. S6).

### UTI ameliorates degradation of endothelial glycocalyx and enhances endothelial barrier function post-cardiac surgery in vitro

**Fig. 3:**
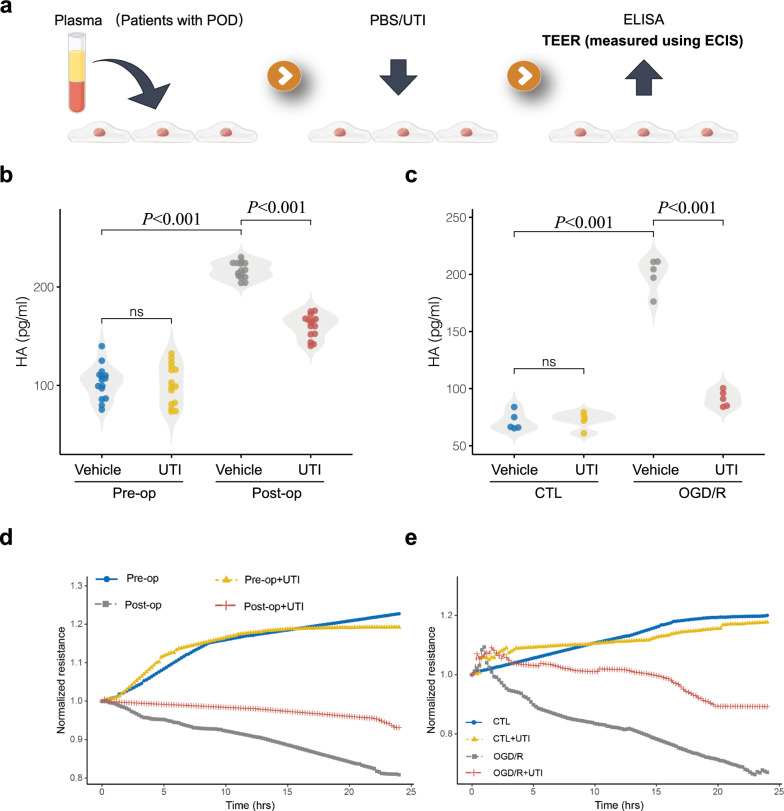
Ulinastatin (UTI) decreases Hyaluronic acid (HA) levels and enhances endothelial barrier function post-cardiac surgery in vitro study. **a** Experimental schematic illustrating the in vitro study design. A pre-post approach was utilized, incorporating both pre-operative and post-operative plasma samples to investigate the effects on HUVECs. Following this, the cells were treated with either PBS or UTI. **b**, **c** HA levels in HUVECs culture medium in various group (n = 5–15). Data shown as Data are presented as median ± interquartile, and statistical significance was determined using one-way ANOVA with Tukey’s post-hoc test or two-tailed log-rank test. (d-e) Trans-endothelial electrical resistance (TER) across confluent HUVECs was monitored over time with an electrical cell-substrate impedance-sensing system

To model the alterations in endothelial barrier function post-cardiac surgery, an in vitro model utilizing plasma from patients with POD was developed (Fig. 3a). Consistent with clinical findings, levels of HA in HUVECs culture medium were significantly elevated in the Post-op group compared to the Pre-op group (median, 217 pg/ml, IQR: 210–224, vs median, 105 pg/ml, IQR: 86–114, *P* < 0.001) (Fig. 3b), indicative of glycocalyx degradation following cardiac surgery. This increase in HA levels corresponded with elevated endothelial permeability in the Post-op group as measured by ECIS (Fig. 3d), suggesting endothelial barrier dysfunction post-surgery. Apparently, treatment with UTI was observed to decrease HA levels (median, 164 pg/ml, IQR: 152–168, vs median, 217 pg/ml, IQR: 210–224, *P* < 0.001) and improve endothelial barrier function when HUVECs were exposed to plasma from POD patients (Fig. 3b, d). Hence, it is inferred that UTI may ameliorate glycocalyx degradation induced by I/R injury from cardiac surgery and enhance endothelial barrier function. Subsequently, an in vitro OGD/R model was established to simulate I/R injury. As expected, UTI treatment was found to mitigate OGD/R-induced glycocalyx degradation and reduce endothelial permeability (Fig. 3c, e). Overall, UTI treatment during cardiac surgery may alleviate glycocalyx degradation, improve endothelial barrier function, and attenuate blood–brain barrier leakage, ultimately potentially preventing POD.

## Discussion

The proteinase inhibitor UTI, recognized for its anti-inflammatory properties, demonstrates promising benefits for patients undergoing cardiac surgery [36]. This study aims to explore the potential role of UTI intraoperative administration on preventing POD among cardiac surgery patients by integrating innovative retrospective study with a conventional prospective observational study and in vitro study. Our research initially identified intraoperative administration of UTI as a protective factor against POD, a conclusion further supported by PSM and machine learning analyses in the retrospective study cohort. Subsequent evaluation in the prospective observational cohort and in vitro study indicated that intraoperative administration of UTI inhibits glycocalyx degradation following cardiac I/R, thus correlating with a diminished risk of POD. Furthermore, our study identified glycocalyx degradation as a potential biomarker for predicting POD in cardiac surgery patients. Therefore, our results suggest that intraoperative administration of UTI may potentially lower the incidence of POD by preserving the integrity of endothelial glycocalyx in patients undergoing cardiac surgery.

Our study highlights the preventive role of UTI in reducing the incidence of delirium after cardiac surgery, particularly in the context of its known anti-inflammatory properties [36, 37]. Previous research has indicated that UTI can reduce the production of pro-inflammatory factors in patients undergoing cardiac surgery [21, 22]. Nevertheless, our study did not find a significant difference in inflammatory markers between patients who received intraoperative UTI treatment and those who did not. This lack of difference may be attributed to more severe injuries in the UTI-treated patients, leading to comparable inflammatory markers between the two groups. Considering the established association between POD and inflammatory responses [16], it suggests that factors beyond inflammation may play a critical role in the reduction of POD following cardiac surgery through the administration of intraoperative UTI. Several studies have shown that BBB dysfunction is linked to the risk of POD [14, 38] and lactate levels [14]. UTI had been demonstrated to ameliorate BBB dysfunction [39] and reduce plasma lactate levels [40]. Another study emphasized that elevated plasma lactate levels one hour after surgery in elderly trauma patients were associated with the occurrence of POD [41]. As anticipated, the current study discovered that increased lactate levels, strongly positively linked with high HA levels, were associated with the probability of POD and could be diminished by UTI. This finding not only illustrates the preventive impact of UTI on POD but also accentuates its potential therapeutic value in mitigating lactate levels. Hence, we hypothesize that UTI could safeguard the integrity of the endothelial glycocalyx, subsequently reducing lactate levels and thereby alleviating BBB disruption caused by cytokines released during cardiac surgery. This might lead to reduced BBB permeability, inhibition of cytokine penetration into brain tissue, and ultimately improvement in neural function.

It is noteworthy that our investigation into glycocalyx degradation post-surgery presents an intriguing finding—it may serve as a predictive biomarker for POD in cardiac surgery patients and could be modulated by intraoperative administration of UTI. Cardiac surgery often triggers a sudden release of inflammatory mediators, such as cytokines and vasoactive peptides [36, 42], into the cardiovascular circulation, mediating endothelial cell injury [43]. As the interface between blood and brain, cerebral endothelium constitutes a major component of the BBB crucial for maintaining BBB integrity [44]. The glycocalyx, a carbohydrate-rich layer lines the endothelium, plays multiple roles in maintaining endothelial cell integrity and vascular homeostasis by preserving barrier function, suppressing inflammation and cell turnover, mediating flow-induced nitric oxide release [45, 46]. Furthermore, inflammatory mediators have been reported to promote the degradation of glycocalyx, endothelial dysfunction, and eventually damage to the BBB, which play crucial roles in the pathogenesis of POD [47, 48]. Protecting against glycocalyx degradation by inhibiting inflammation represents a promising therapeutic approach [49]. Studies have indicated that glycocalyx degradation in surgical patients is associated with the occurrence of POD and may contribute to its development [50, 51]. A study demonstrated that perioperative administration of UTI inhibit serine proteases and hyaluronidase to reduce glycocalyx degradation [52]. Thus, as a broad-spectrum protease inhibitor, UTI may counter hyaluronidase, ameliorate glycocalyx degradation, enhance endothelial cell barrier function, mitigate BBB permeability, diminish inflammation-induced neuronal injury, and consequently mitigate the onset of POD. The subsequent in vitro study demonstrated that UTI treatment effectively mitigated glycocalyx degradation, characterized by reduced HA levels, and enhanced endothelial barrier function in an in vitro model utilizing plasma from POD patients. Furthermore, UTI’s protective effect was evidenced by the mitigation of glycocalyx degradation and reduction of endothelial permeability in an in vitro model simulating I/R injury. These findings imply that UTI administration during cardiac surgery could be crucial in preserving glycocalyx integrity, enhancing endothelial barrier function, and potentially reducing blood–brain barrier permeability, offering a promising approach for POD prevention.

By exploring the potential correlations between glycocalyx degradation and plasma lactate levels, we have uncovered a new dimension in our understanding of POD pathophysiology. Elevated plasma lactate levels, often indicative of tissue hypoperfusion and cellular injury, have been linked to POD occurrence, suggesting a complex interplay between metabolic disturbances and cognitive outcomes. Therefore, the reduction in plasma lactate levels resulting from UTI administration not only alleviates its potential detrimental effects on neuronal function but also underscores the importance of metabolic homeostasis in preserving cognitive health. By inhibiting endothelial glycocalyx degradation and preserving BBB function, UTI could potentially reduce lactate levels, protect neurons from reperfusion injury-related cytokine and improve neurovascular outcomes.

This study has several strengths, such as its combination of a multi-center retrospective approach with a single-center prospective cohort design, along with in vitro studies. However, it’s important to acknowledge certain limitations. While we are aware that electronic medical records have inherent limitations, we took extra measures to ensure the validity of our results. In the subsequent validation cohort, we carried out thorough follow-ups to improve the accuracy and reliability of our findings. Despite using univariate and multivariate logistic regression and PSM to adjust for confounding factors, we could not completely eliminate the potential for selection bias and residual confounding in the retrospective cohort. Additionally, the lower incidence of POD observed in our study compared to previous studies may be influenced by differences in patient characteristics, surgical procedures, retrospective nature of the study and the prophylactic use of dexmedetomidine among all cardiac surgery patients. This discrepancy could also be since our study was not a randomized controlled trial and may not have captured all relevant factors contributing to POD. Therefore, future research using robust designs, like qualitative data collection, longitudinal studies, and multi-center randomized clinical trials, is necessary to confirm these findings. Additionally, a deeper investigation into the pathophysiological mechanisms behind the protective effects of UTI on glycocalyx degradation is essential to advance our understanding in this area.

## Conclusion

In summary, our research sheds light on the potential benefits of intraoperative administration of UTI in reducing POD among cardiac surgery patients through the preservation of endothelial glycocalyx integrity. The findings emphasize the importance of mitigating endothelial glycocalyx degradation in POD preventing. This study enhances our comprehension of the pathophysiology of POD and underscores the practical implications of utilizing UTI as a preventative strategy to enhance cognitive outcomes in cardiac surgery patients.

## Supplementary Information

Additional file1 (PDF 2033 KB)

## Abbreviations

UTI: Ulinastatin
ATCC: American type culture collection
AUC: Area under the receiver operating characteristic curve
BBB: Blood–brain barrier
CAM: Confusion assessment method
CAM-ICU: Confusion assessment method for the intensive care unit
CPB: Cardiopulmonary bypass
DCA: Decision curve analysis
DTC: Decision tree classifier
FBS: Fetal bovine serum
GBC: Gradient boosting classifier
GNB: Gaussian Naive Bayes
HA: Hyaluronic acid
I/R: Ischemia–reperfusion
IQR: Interquartile range
LASSO: Least absolute shrinkage and selection operator
LOWESS: Locally weighted scatterplot smoothing
MFT: Multiple frequency/time
OGD/R: Oxygen–glucose deprivation/reoxygenation
OR: Odds ratio
PBS: Phosphate-buffered saline
POD: Postoperative delirium
PSM: Propensity score matching
RCS: Restricted cubic splines
ROC: Receiver operating characteristic
RF: Random forest
SHAP: SHapley Additive exPlanations
TEER: Transendothelial electrical resistance

## Declarations

### Availability of data and materials

On reasonable request, data, analytic methods, and study materials will be made available from the corresponding authors to other researchers for purposes of reproducing the results or replicating the procedure.
