# Supplementary material for: Ulinastatin treatment mitigates glycocalyx degradation and associated with lower postoperative delirium risk in patients undergoing cardiac surgery: a multicentre observational study
Source: Crit Care. 2025 Jan 29;29:52. doi: 10.1186/s13054-025-05296-9 (PMC11776223; doi:10.1186/s13054-025-05296-9)
Supplement: Supplementary file 1 — Additional file1 (PDF 2033 KB) [file 13054_2025_5296_MOESM1_ESM.pdf]

Supplementary material

Ulinastatin treatment Mitigates Glycocalyx Degradation and associated with Lower Postoperative Delirium Risk in Patients Undergoing Cardiac Surgery: A Multicentre Observational Study

Xiao Ran[1,2#], Tingting Xu [1,2#], Jieqiong Liu[3], Shaobing Yang [3], Fang Luo[3], Rongxue Wu[4], Juan Tan[3], Hang Ruan[1,2*], Qin Zhang[3*]

[1] Department of Critical Care Medicine, Tongji Hospital, Tongji Medical College, Huazhong University of Science and Technology, Wuhan, China.

[2] Department of Emergency Medicine, Tongji Hospital, Tongji Medical College, Huazhong University of Science and Technology, Wuhan, China.

[3] Department of Anesthesiology, Hubei Key Laboratory of Geriatric Anesthesia and Perioperative Brain Health, and Wuhan Clinical Research Center for Geriatric Anesthesia, Tongji Hospital, Tongji Medical College, Huazhong University of Science and Technology, Wuhan, 430030, China

[4] Department of Biological Sciences Division - Cardiology, University of Chicago

[#] These authors contributed equally to this work.

*Corresponding authors:

Qin Zhang: 1095# Jiefang Ave, Tongji Hospital, Tongji Medical College, Huazhong University of Science and Technology, Wuhan, 430000, *China*. Phone: +86-15717554768; Email: qzhang8@tjh.tjmu.cn (https://orcid.org/0000-0002-5664-5823)

Hang Ruan: 1095# Jiefang Ave, Tongji Hospital, Tongji Medical College, Huazhong University of Science and Technology, Wuhan, 430000, *China*. Email: dr.ruanhang@139.com (https://orcid.org/0000-0001-7738-0177)

Delirium Diagnostic Criteria

The adapted Diagnostic and Statistical Manual of Mental Disorders, Fifth Edition (DSM-5) scale**[3]**.

(a) Disturbance: i) Attention: Reduced ability to direct, focus, sustain, and shift attention. From: 20-1, MOyB (if completed), comments such as “distractible” or “inattentive” ii) Awareness: Reduced orientation to the environment. From: Comments indicating “drowsy” or “agitated”

(b) Disturbance Characteristics: Develops over a short period (hours to days); Represents a change from baseline attention and awareness; Tends to fluctuate in severity throughout the day. From: Documentation as a new issue by medical staff or expressed concerns

(c) Additional Cognitive Disturbance: (e.g., memory deficit, disorientation, language impairment, visuospatial issues, or perceptual changes)

(d) Exclusions: The disturbance in Criteria A and C are: i) Better explained by an existing neurocognitive disorder, ii) Occurs in the context of severely reduced arousal like coma From: History suggesting a progressive or severely obtunded patient

(e) Physiological Consequence Evidence: The disturbance is a direct physiological consequence of a medical condition, substance intoxication or withdrawal, toxin exposure, or multiple causes From: Documentation of acute illness or contributing factors

Probable Delirium Diagnosis: All items a, b, c, and e ‘yes’, plus d ‘no’

Possible Delirium Diagnosis: If any “?” or e ‘no’

Correlation Analysis

In step 2 of this study, the correlation between POD and HA concentration was analyzed using locally weighted scatterplot smoothing (LOWESS) regression. Restricted cubic splines (RCS) regression and logistic regression were also used to further analyze the association between POD and HA concentration. Pearson correlation coefficients was used to assess the strength and direction of the linear relationship between the variables. The linear relationship between HA concentration and lactate level was analyzed using scatter fit-slope fitting. Logistic regression analysis evaluated the correlation between intraoperative administration of UTI and POD, adjusted for preoperative HA, preoperative troponin I and preoperative lactate dehydrogenase in the prospective observational cohort. Collinearity analysis was conducted before multivariate logistic regression analysis. The sensitivity and specificity of HA concentration, lactate, inflammatory markers and biochemical indicators in predicting POD were calculated using receiver operating characteristic (ROC) curves.

Sample Size

According to the literature, the incidence of POD in the UTI treatment was reported as 11%, while in the non-UTI treatment, it was 30.8%[1, 2]. The sample size for both groups was calculated using PASS 15.0 software at $\alpha = 0.05$ (two-sided) and $\beta = 0.10$, requiring the inclusion of 174 patients. Accounting for a 20% attrition rate, the estimated minimum sample size was 218.

Table S1. Collinearity diagnostics of variables for multivariate analysis.

Variable	VIF	Tolerance
Age	1.200	0.831
Weight	1.750	0.571
Height	2.540	0.393
Gender	2.070	0.483
Hypertension	1.190	0.840
Diabetes mellitus	1.070	0.930
Respiratory diseases	1.000	0.997
Renal dysfunction	1.030	0.967
Tumour	1.010	0.992
History of stroke	1.020	0.976
History of surgical	1.010	0.986
Shock	1.010	0.991
Infection	1.090	0.914
Anemia	1.050	0.951
Cardiac surgery with CPB	1.140	0.875
UTI treatment	1.020	0.976

Abbreviations: VIF, variance inflation factor; CPB, cardiopulmonary bypass; UTI, ulinastatin.

Table S2. Participants' baseline characteristics and outcome of the retrospective cohort after propensity score matching.

Characteristics	Total (n=5296)	UTI treatment (n=2648)	Non-UTI treatment (n=2648)	*P*-value
Age, years	60 (52-68)	61 (53-68)	60 (51-68)	0.127
Weight, kg	63 (55-71)	63 (55-71)	63 (55-71)	0.626
Height, cm	166 (160-170)	166 (160-170)	166 (160-170)	0.771
Gender, n (%)				
Male	3251 (61.4)	1608 (60.7)	1643 (62.0)	0.323
Female	2045 (38.6)	1040 (39.3)	1005 (38.0)	
Chronic comorbidities, n (%)				
Hypertension	1688 (31.9)	793 (29.9)	895 (33.8)	0.003
Diabetes mellitus	378 (7.1)	183 (6.9)	195 (7.4)	0.522
Respiratory diseases	31 (0.6)	18 (0.7)	13 (0.5)	0.368
Renal dysfunction	60 (1.1)	27 (1.0)	33 (1.2)	0.436
Tumour	46 (0.9)	21 (0.8)	25 (0.9)	0.554
History of stroke	159 (3.0)	80 (3.0)	79 (3.0)	0.936
History of surgical	1185 (22.4)	598 (22.6)	587 (22.2)	0.717
Preoperative				
Shock, n (%)	57 (1.1)	31 (1.2)	26 (1.0)	0.506
Infection, n (%)	1386 (26.2)	705 (26.6)	681 (25.7)	0.453
Anemia, n (%)	1601 (30.2)	778 (29.4)	823 (31.1)	0.178
Cardiac surgery with CPB, n (%)	4388 (82.9)	2190 (82.7)	2198 (83.0)	0.771
Primary outcome				
POD, n (%)	703 (13.3)	283 (10.7)	420 (15.9)	<0.001

Continuous variables were presented as median and interquartile range. Categorical variables were reported as number (proportions). Abbreviations: UTI, ulinastatin; CPB, cardiopulmonary bypass; POD, postoperative delirium.

Table S3. Demographics, clinical characteristics, and outcomes of cardiac surgery patients in the prospective observational cohort.

Characteristics	Total (n=241)	UTI treatment (n=99)	Non-UTI treatment (n=142)	*P*-value
Age, years	56 (47-62)	55 (46-62)	56 (48-63)	0.446
Weight, kg	62 (55-73)	62 (53-73)	62 (55-73)	0.637
Height, cm	167 (160-170)	166 (160-170)	168 (160-171)	0.666
Gender, n (%)				
Female	109 (45.2)	47 (47.5)	62 (43.7)	0.558
Male	132 (54.8)	52 (52.5)	80 (56.3)	
Chronic comorbidities, n (%)				
Hypertension	114 (47.3)	49 (49.5)	65 (45.8)	0.569
Diabetes mellitus	22 (9.1)	11 (11.1)	11 (7.7)	0.372
Respiratory diseases	2 (0.8)	1 (1.0)	1 (0.7)	1.000
Renal dysfunction	7 (2.9)	4 (4.0)	3 (2.1)	0.450
Tumour	9 (3.7)	3 (3.0)	6 (4.2)	0.740
History of stroke	16 (6.6)	10 (10.1)	6 (4.2)	0.071
Preoperative				
cTNI, pg/mL	7.4 (2.7-25.4)	6.4 (2.2-18.0)	8.2 (3.3-37.4)	0.048
BNP, pg/mL	296.0 (80.1-1194.0)	280.3 (61.1-953.0)	300.2 (87.6-1236.0)	0.137
WBC, ×10^9/L	6.33 (5.20-7.68)	6.27 (5.19-7.70)	6.36 (5.20-7.68)	0.710
LDH, U/L	206 (175-245)	195 (166-225)	215 (180-250)	0.004
Hb, g/L	130 (119-144)	130 (122-144)	131 (118-144)	0.644
Preoperative HA, pg/mL	291 (244-341)	305 (273-342)	282 (231-341)	0.048
Primary outcome				
POD, n (%)	30 (12.4)	7 (7.1)	23 (16.2)	0.035
Secondary outcomes				
Lac, mmol/L	2.8 (1.6-4.5)	1.8 (1.3-3.0)	3.2 (2.3-5.4)	<0.001
Postoperative HA, pg/mL	604 (517-692)	530 (478-610)	644 (567-711)	<0.001
HA concentration difference, pg/mL	312 (217-392)	236 (161-320)	364 (281-428)	<0.001
Duration of surgery, min	326 (265-398)	308 (268-380)	330 (265-415)	0.203
Hospital length of stay, days	21 (17-27)	22 (17-27)	20 (17-27)	0.542

Continuous variables were presented as median and interquartile range. Categorical variables were reported as number (proportions). Abbreviations: UTI, ulinastatin; cTNI, cardiac troponin I; BNP, B-type natriuretic peptide; WBC, white blood cells; LDH, lactate dehydrogenase; Hb, hemoglobin; HA, hyaluronic acid; POD, postoperative delirium; Lac, lactate.

Table S4. Collinearity diagnostics of variables for multivariate analysis.

Variable	VIF	Tolerance
Age	1.420	0.704
Weight	2.170	0.461
Height	3.080	0.325
Gender	2.440	0.410
Hypertension	1.360	0.737
Diabetes mellitus	1.110	0.902
Respiratory diseases	1.160	0.860
Renal dysfunction	1.440	0.695
Tumour	1.100	0.912
History of stroke	1.240	0.809
Preoperative		
cTNI	1.080	0.925
BNP	1.310	0.763
WBC	1.510	0.662
LDH	1.400	0.713
Hb	1.450	0.692
Preoperative HA	1.020	0.977

Abbreviations: VIF, variance inflation factor; cTNI, cardiac troponin I; BNP, B-type natriuretic peptide; WBC, white blood cells; LDH, lactate dehydrogenase; Hb, hemoglobin; HA, hyaluronic acid.

Table S5. The mediation effect analysis of the relationship between intraoperative administration of ulinastatin, hyaluronic acid concentration difference, and postoperative delirium.

POD	Coefficient	Std.err.	z	95%CI	*P* value
Intraoperative administration of UTI					
Total effect	-0.103	0.046	-2.21	-0.194--0.012	0.027
Indirect effect	-0.009	0.050	-0.18	-0.106-0.088	0.855
Direct effect	-0.094				

Abbreviations: POD, postoperative delirium; UTI, ulinastatin.

Table S6. Correcting the mediation effects model for age and sex.

POD	Coefficient	Std.err.	z	95%CI	*P* value
Intraoperative administration of UTI					
Total effect	-0.100	0.046	-2.170	-0.190--0.009	0.030
Indirect effect	-0.006	0.049	-0.120	-0.103-0.091	0.903
Direct effect	-0.094	.	.		

Abbreviations: POD, postoperative delirium; UTI, ulinastatin.

Table S7. Correcting the mediation effects model for age, sex, height and weight.

POD	Coefficient	Std.err.	z	95%CI	*P* value
Intraoperative administration of UTI					
Total effect	-0.099	0.046	-2.140	-0.189--0.008	0.032
Indirect effect	-0.005	0.049	-0.100	-0.102-0.092	0.918
Direct effect	-0.094	.	.		

Abbreviations: POD, postoperative delirium; UTI, ulinastatin.

Table S8. Correcting the mediation effects model for age, sex, height, weight, Diabetes mellitus and Hypertension.

POD	Coefficient	Std.err.	z	95%CI	*P* value
Intraoperative administration of UTI					
Total effect	-0.097	0.046	-2.110	-0.186--0.007	0.035
Indirect effect	-0.010	0.049	-0.210	-0.107-0.086	0.836
Direct effect	-0.086	.	.		

Abbreviations: POD, postoperative delirium; UTI, ulinastatin.

Table S9. The mediation effect analysis of the relationship between intraoperative administration of ulinastatin, hyaluronic acid concentration difference, and postoperative delirium (medeff).

Effect	Mean	95% Conf. Interval
Total effect	-0.097	-0.174--0.011
Indirect effect	-0.086	-0.142--0.043
Direct effect	-0.006	-0.093-0.093

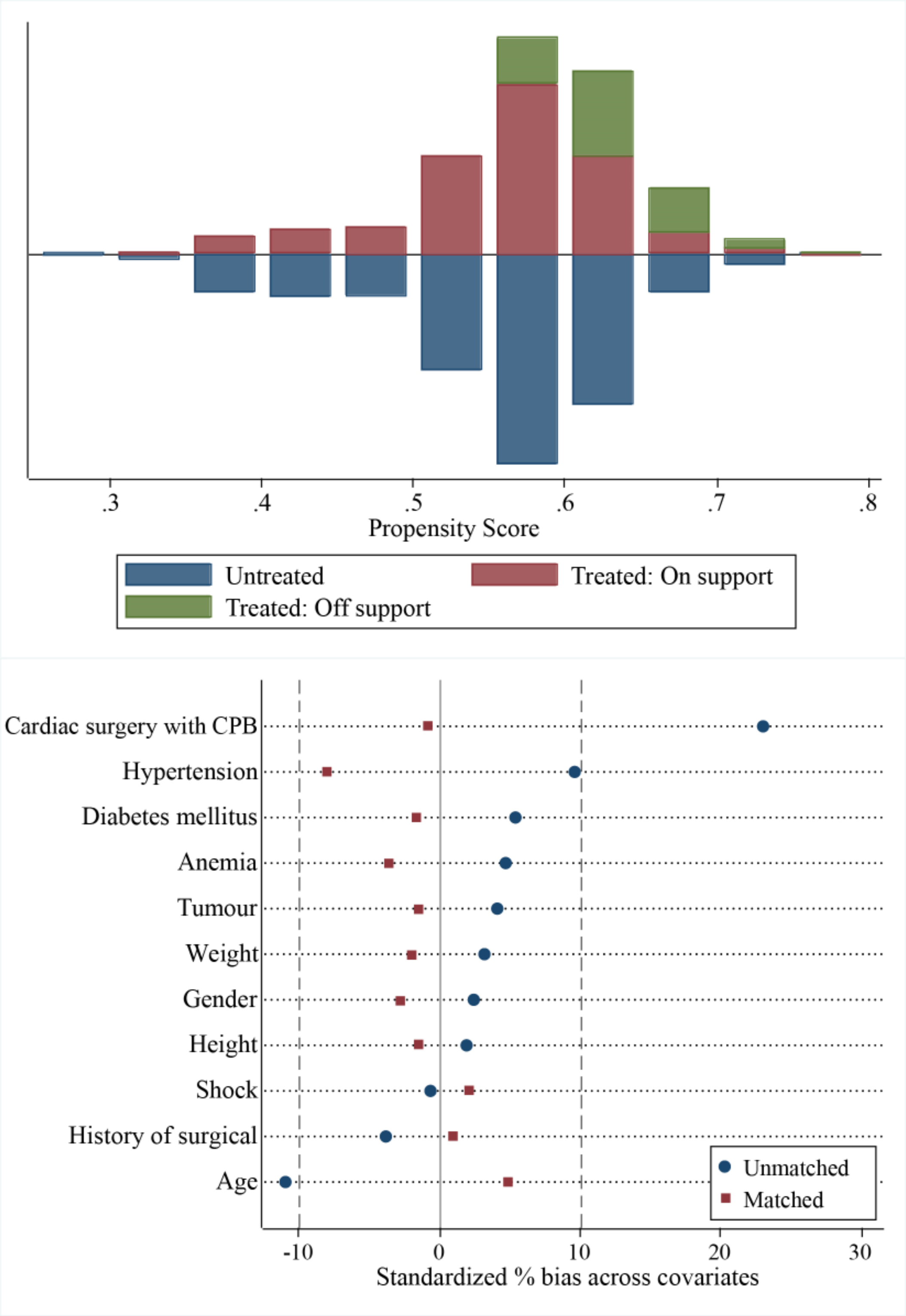

Figure S1: Changes in variables before and after performing propensity score matching. Abbreviations: CPB, cardiopulmonary bypass

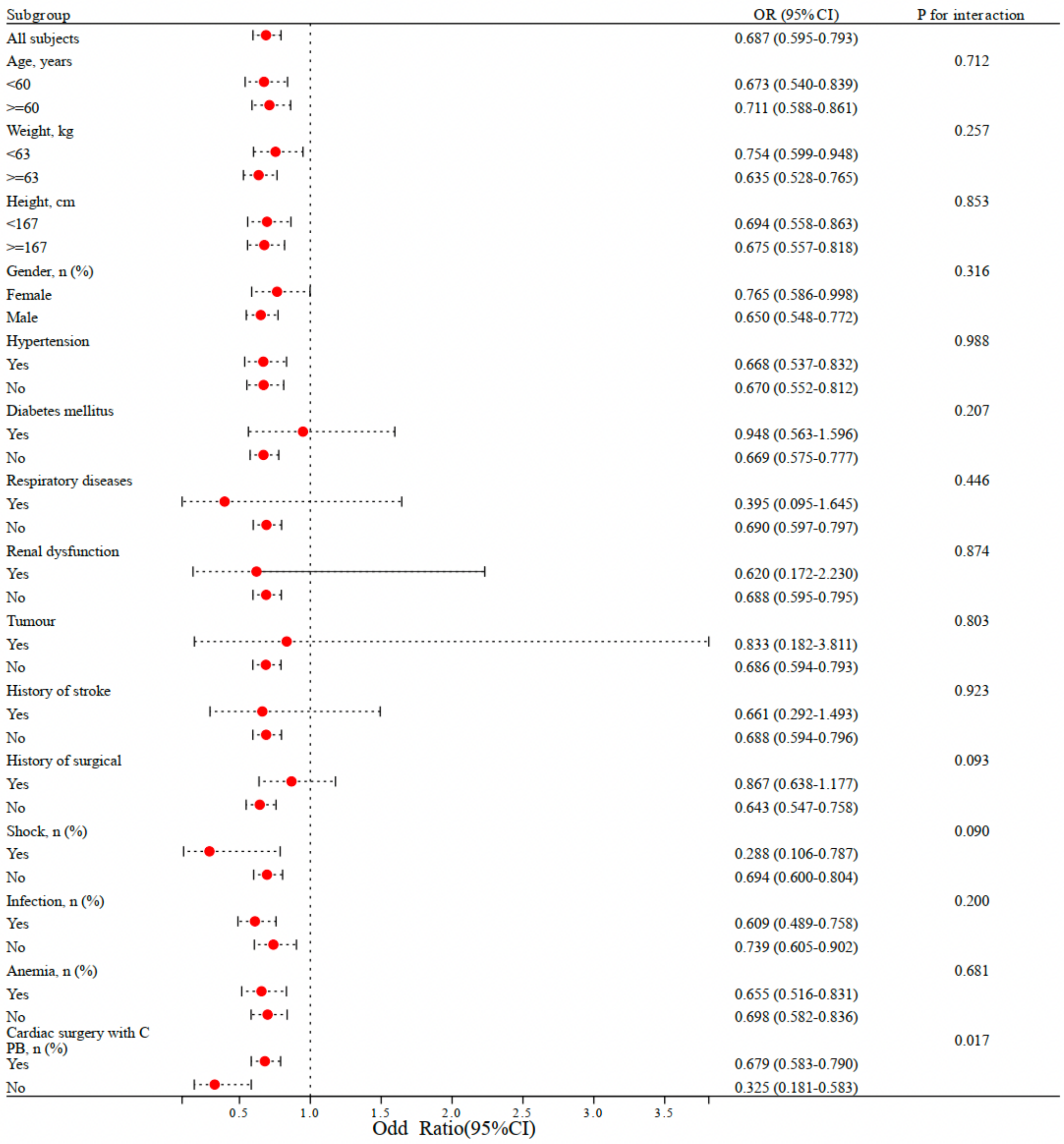

Figure S2: Effect of intraoperative administration of ulinastatin on POD stratified by variables: a forest plot analysis. In the detailed subgroup analyses, intraoperative UTI treament was found to be associated with a reduced risk of POD, especially in cardiac surgery patients undergoing CPB (*P* for interacion = 0.017).

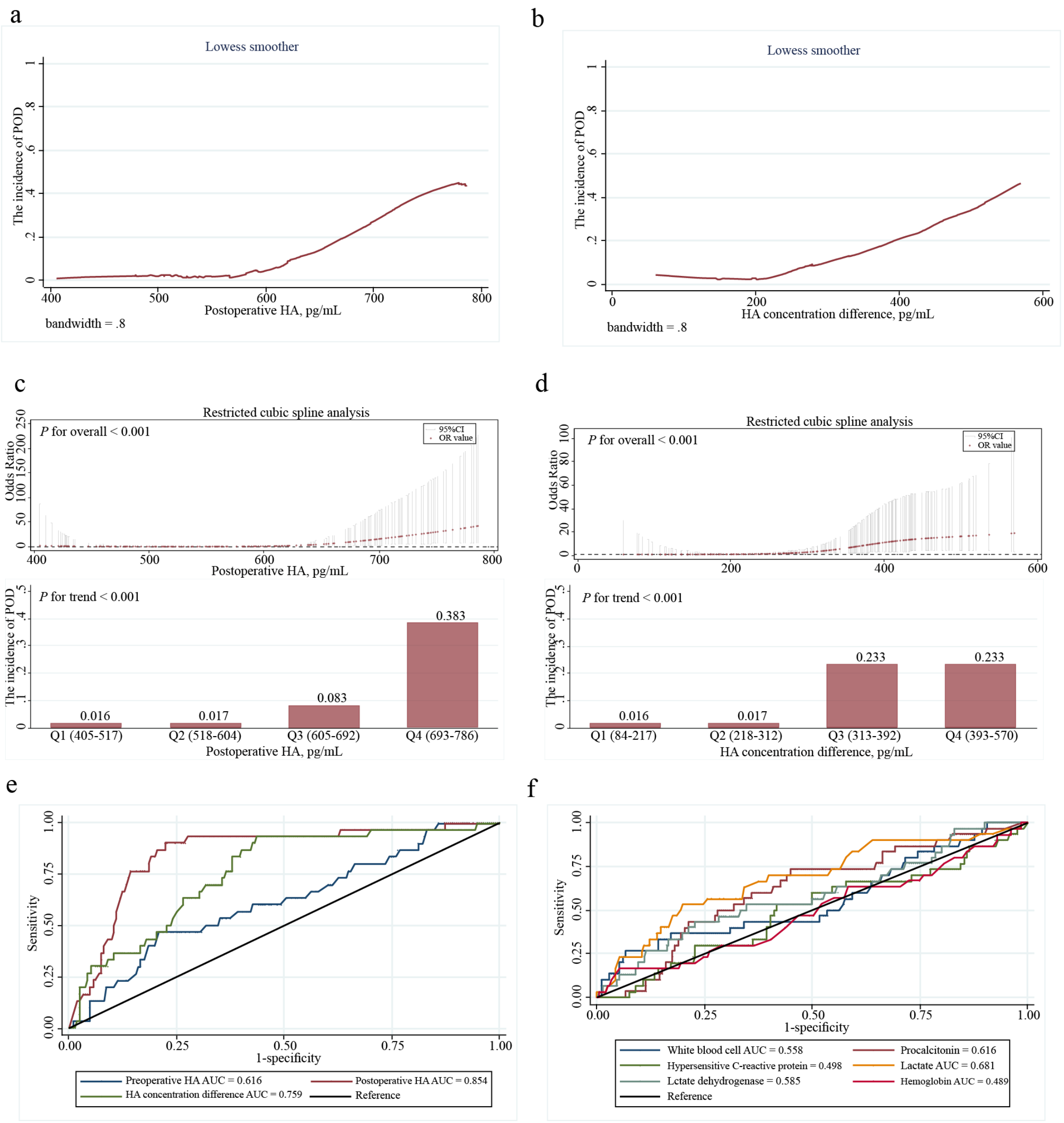

Figure S3: (a) LOWESS regression curve representing the non-linear relationship between postoperative HA and POD. (b) LOWESS regression curve representing the non-linear relationship between HA concentration difference and POD. (c) RCS regression curve illustrating the non-linear relationship between postoperative HA and POD (*P* for overall<0.001). (d) RCS regression curve illustrating the non-linear relationship between HA concentration difference and POD (*P* for overall<0.001). (e) ROC curves of preoperative HA, postoperative HA, and HA concentration difference for predicting POD. (f) ROC curves of postoperative lactate, white blood cells, procalcitonin, hemoglobin, lactate dehydrogenase, and hypersensitivity C-reactive protein for predicting POD.

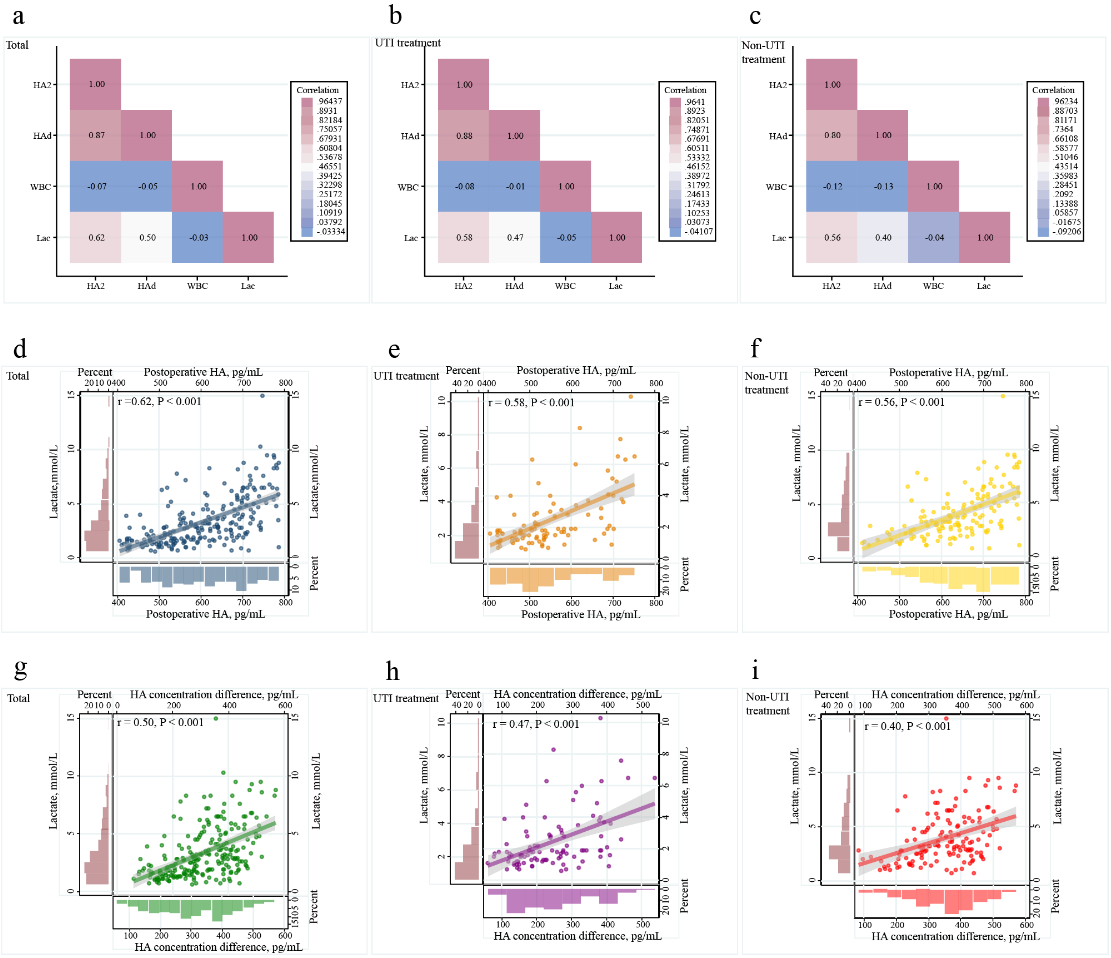

Figure S4. Correlation analysis between variables. Abbreviations: UTI, ulinastatin; HA, hyaluronic acid; HA2, postoperative HA; HAd, HA concentration difference; WBC, white blood cells; Lac, lactate. The Pearson correlation coefficient analysis demonstrated a strong association between HA concentration and postoperative lactate levels (Figure 3a-c). Additionally, the scatter fit-slope fitting method illustrated a positive linear relationship between HA concentration and postoperative lactate levels (Figure 3d-i). Taken together, these findings suggest that intraoperative UTI treatment may reduce the occurrence of POD by inhibiting glycocalyx degradation and potentially lower postoperative lactate levels in patients undergoing cardiac surgery.

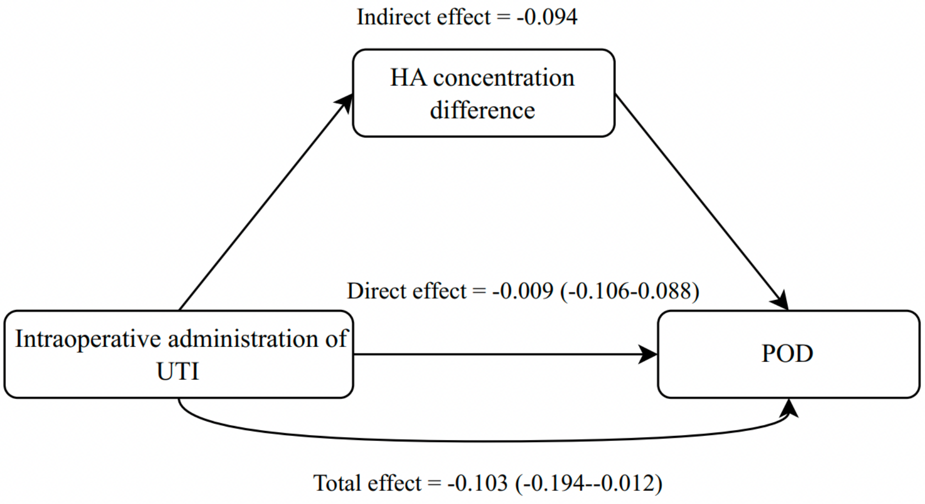

Figure S5: The mediation effect analysis of the relationship between intraoperative administration of ulinastatin, hyaluronic acid concentration difference, and postoperative delirium. This figure showed that the influence of intraoperative UTI treatment on reducing POD was partially mediated by its capacity to lower HA levels.

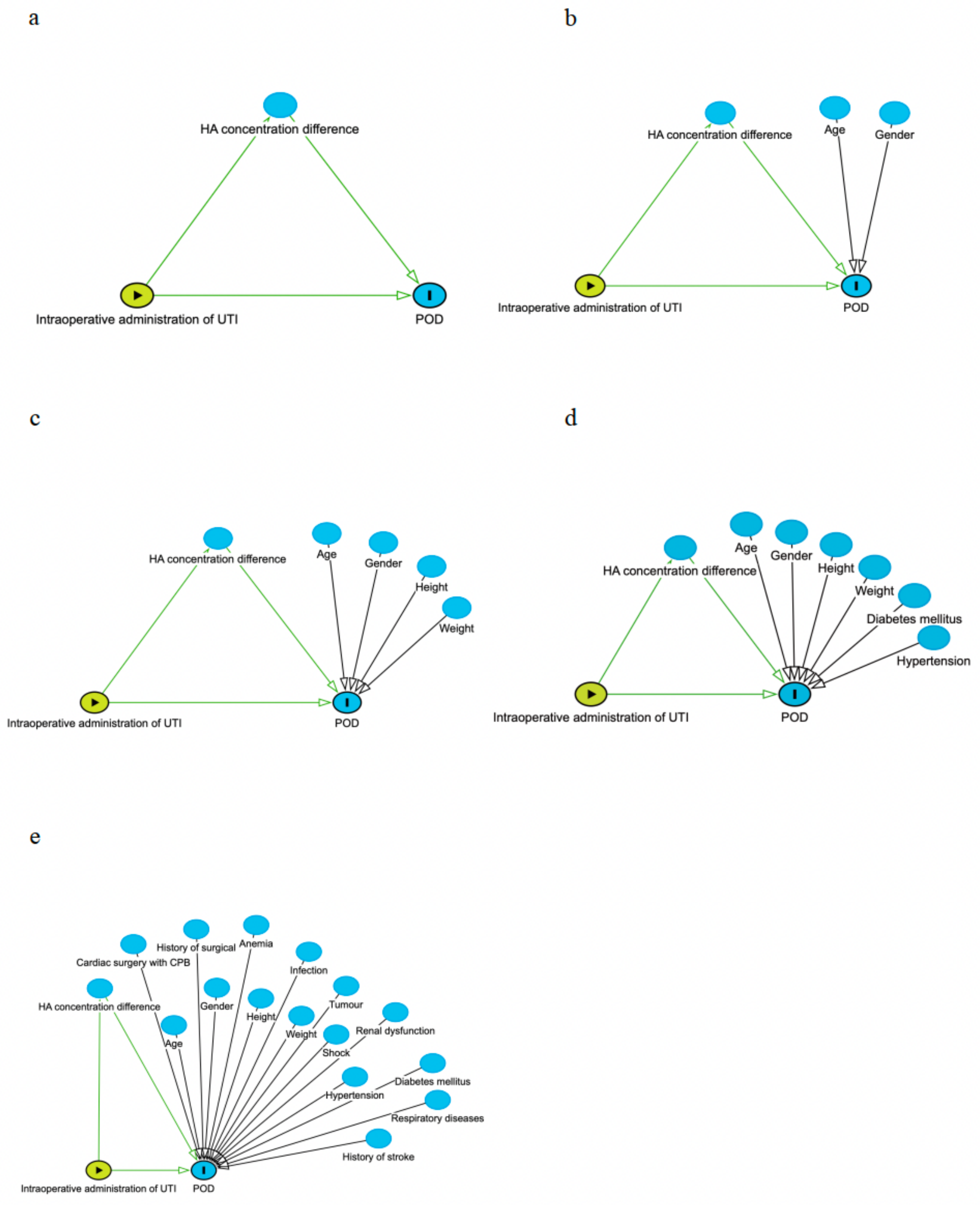

Figure S6: The Directed Acyclic Graph (DAG) diagram of mediation effect. The DAG clearly illustrated the indirect effect of the intraoperative administration of UTI on the POD hrough the HA concentration difference, while also depicting the direct effect of the intraoperative administration of UTI on the POD.

Reference:

1. Lv ZT, Huang JM, Zhang JM, Zhang JM, Guo JF, Chen AM: **Effect of Ulinastatin in the Treatment of Postperative Cognitive Dysfunction: Review of Current Literature**. *Biomed Res Int* 2016, **2016**:2571080.

2. Duan M, Liu F, Fu H, Feng S, Wang X, Wang T: **Effect of Ulinastatin on Early Postoperative Cognitive Dysfunction in Elderly Patients Undergoing Surgery: A Systemic Review and Meta-Analysis**. *Front Neurosci* 2021, **15**:618589.

3. **Retrospective delirium ascertainment from case notes: a retrospective cohort study**. *BMJ Open* 2021, **11**(5):e042440.
